# Case Report: Candidate Genes Associated With Prenatal Ultrasound Anomalies in a Fetus With Prenatally Detected 1q23.3q31.2 Deletion

**DOI:** 10.3389/fgene.2021.696624

**Published:** 2021-09-23

**Authors:** Jiahao Song, Qian Zhang, Bing Lu, Zhongshan Gou, Ting Wang, Hui Tang, Jingjing Xiang, Wei Jiang, Xuedong Deng

**Affiliations:** ^1^Center for Medical Ultrasound, The Affiliated Suzhou Hospital of Nanjing Medical University, Suzhou, China; ^2^Department of Obstetrics and Gynecology, The Affiliated Suzhou Hospital Nanjing Medical University, Suzhou, China; ^3^Center for Reproduction and Genetics, The Affiliated Suzhou Hospital Nanjing Medical University, Suzhou, China

**Keywords:** unroofed coronary sinus syndrome, persistent left superior vena cava, chromosomal microarray analysis, kidney, ultrasound, fetal growth restriction

## Abstract

**Background:** Patients with deletions involving the long arm of chromosome 1 are rare, and the main aim of this study was to refine the genotype-phenotype correlation.

**Case Report:** In this report, a 28-year-old pregnant woman, gravida 2 para 1, at 25^+4^ weeks of gestation underwent ultrasound examination in our institute. The ultrasonographic findings of the fetus were as follows: (1) fetal growth restriction; (2) cleft lip and palate; (3) bilateral renal hypoplasia; (4) lateral ventriculomegaly; (5) single umbilical artery; (6) absent stomach; (7) coronary sinus dilatation with persistent left superior vena cava, ventricular septal defect and unroofed coronary sinus syndrome. Chromosomal microarray analysis of amniotic fluid from the fetus revealed a 28.025 Mb deletion in 1q23.3q31.2, spanning from position 164,559,675 to 192,584,768 (hg19).

**Conclusion:** Genotype-phenotype correlation might improve prenatal diagnosis of fetuses with chromosome 1q deletion. *PBX1* could be a candidate gene for fetal growth restriction, renal hypoplasia and congenital heart disease. Fetal growth restriction was accompanied by decreased renal volume in the fetus. Combined with ultrasonic examination, the application of chromosomal microarray analysis will provide accurate prenatal diagnosis.

## Introduction

The incidence of chromosome 1q deletion in the population has not been reported due to the limited number of reported cases. In this study, we retrospectively reviewed a cohort study of 4,119 pregnancies from September 2015 to December 2020, and only one case was detected (0.024%). Available data on these patients with the proximal and intermediate deletions on chromosome 1q, indicate that the most common clinical features include fetal growth restriction, microcephaly, intellectual disability, abnormal ears, palmprint abnormality, fifth finger clinodactyly, fingernail dysplasia, multiple hernia, short limbs and external genital malformations (Scarbrough et al., 1988). In recent years, the application of chromosomal microarray analysis (CMA) makes it possible to identify more fetuses with chromosomal abnormalities. CMA is a high-resolution technique that can detect aneuploidy, microduplication and microdeletions throughout the genome (Xiang et al., 2020). Overall, the literature clearly shows that CMA will detect clinically significant submicroscopic CNVs other than karyotypes in about 6–7% of fetuses with structural anomalies observed by ultrasound (Vora et al., 2016). Based on this, the American Congress of Obstetricians and Gynecologists (ACOG) now recommends CMA as the first tier approach for the diagnosis of fetal structural abnormalities (Levy and Wapner, 2018).

## Case Presentation

**Figure 1:**
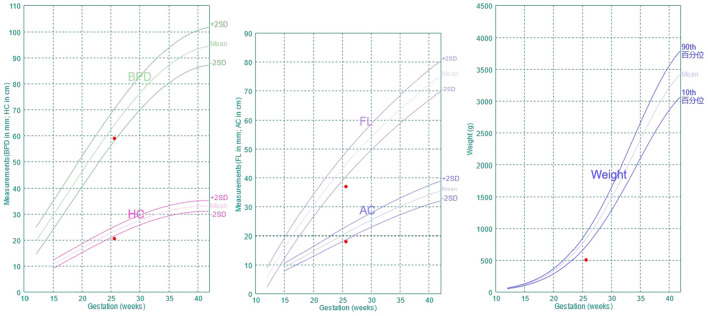
Fetal growth curve.

A 28-year-old pregnant woman, gravida 2 para 1, at 25^+4^ weeks of gestation underwent ultrasound examination in our institute. The biparietal diameter was 5.9 cm (−1.67SD), the head circumference was 20.6 cm (−3.11SD), the abdominal circumference was 18.1 cm (−2.55SD) and the length of femur was 3.7 cm (−4SD) (Figure 1). The results indicated that all the biological measurement indexes were consistent with the ultrasound findings of 22^+4^ weeks of pregnancy. The size of left and right kidneys was small (left: 2.1 × 1.1 × 0.9 cm, right: 1.8 × 1.0 × 1.0 cm).

**Figure 2:**
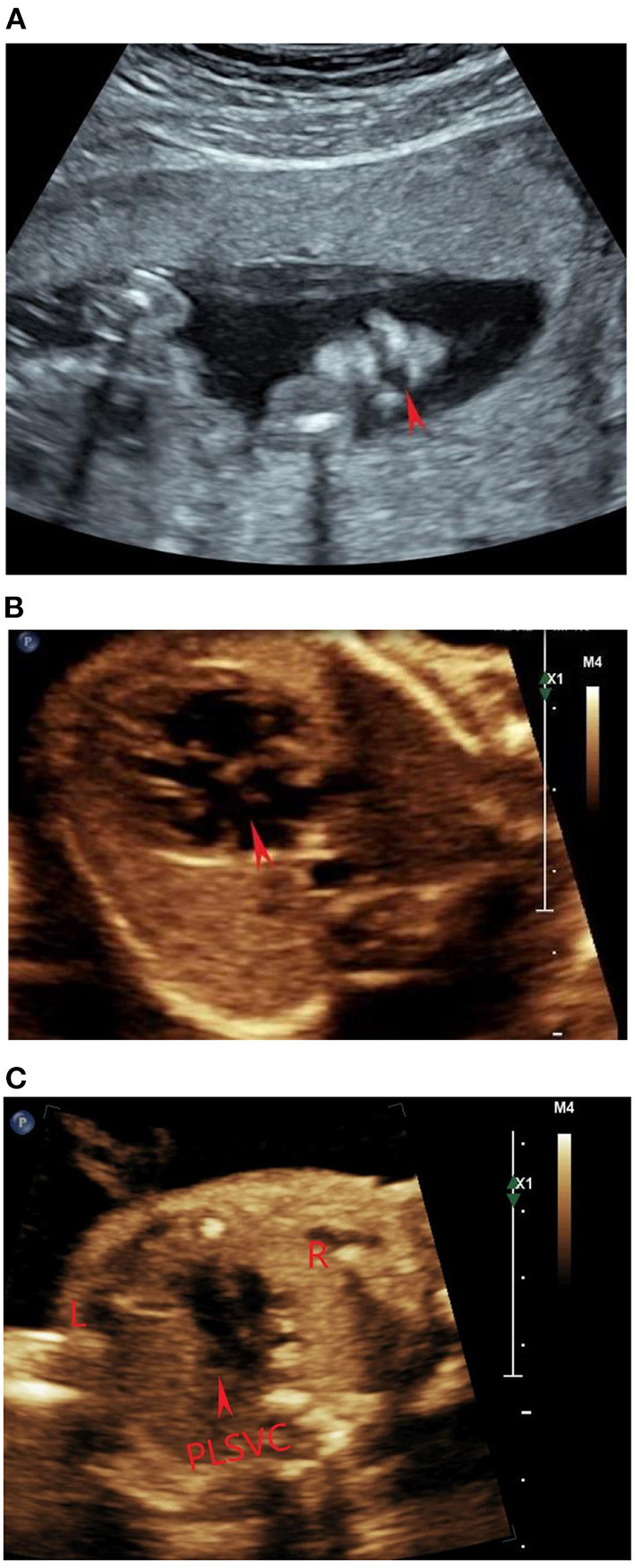
**(A)** Transabdominal scan performed at 25 weeks 4 days in coronal plane: the cleft lip appeared as an anhecogenic area at the level of the left upper lip. (**B**) Transverse section of fetal chest at 25 weeks 4 days: the red arrow pointed to the defect between the left atrium (LA) and the coronary sinus (CS). (**C**) The three-vessel and tracheal (3VT) view at the upper mediastinum showed a supernumerary vessel to the left of the pulmonary trunk and arterial duct. The red arrow pointed to the persistent left superior vena cava (PLSVC) draining into the right atrium.

**Figure 3:**
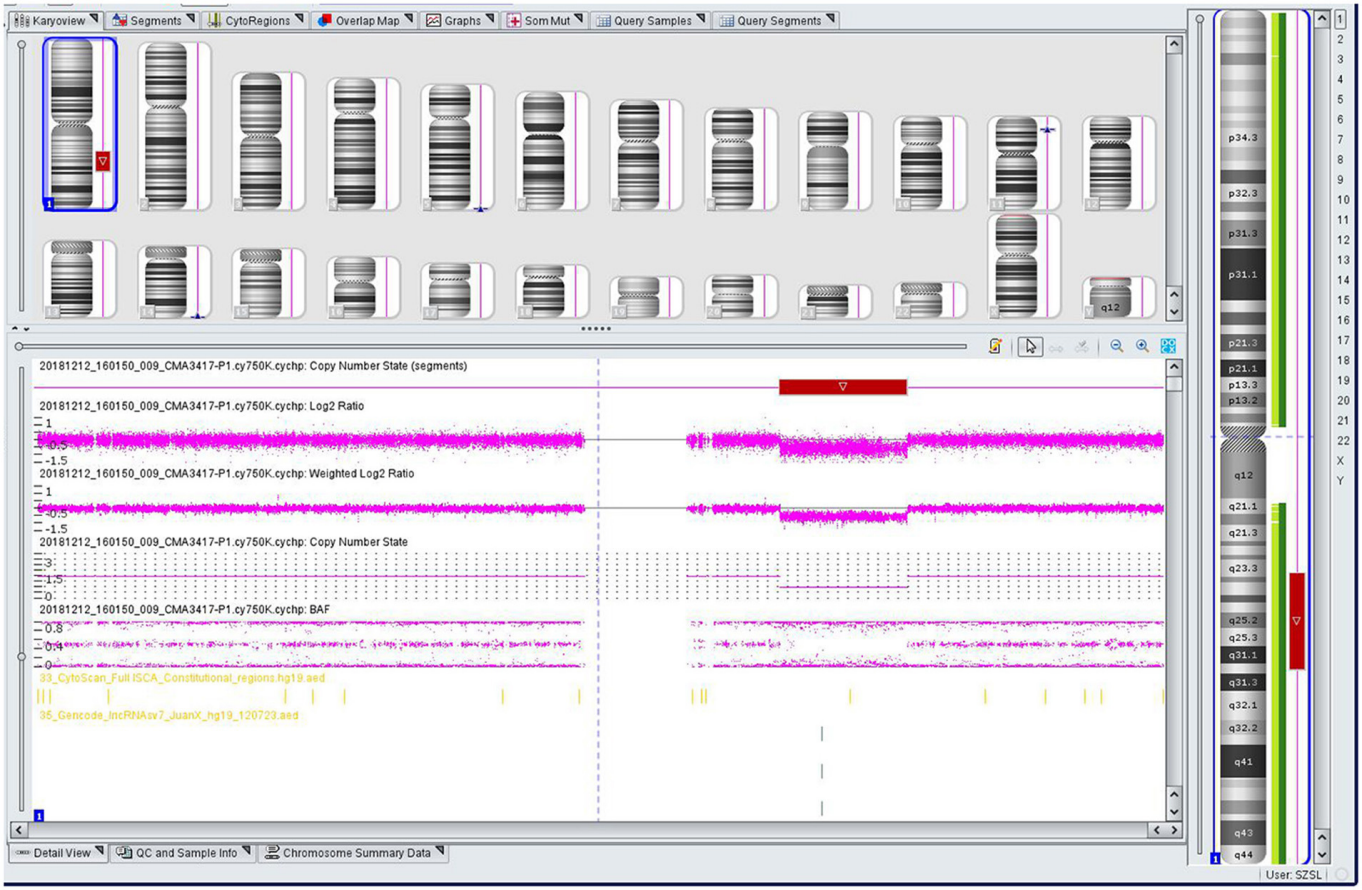
Single nucleotide polymorphism (SNP) array results of the fetus: the red rectangle showed the deletion region of 1q23.3q31.2.

The ultrasound machines used in this study were a Voluson E10 (GE Healthcare Ultrasound, Milwaukee, WI, USA) with a 1–5-MHz transabdominal 2D curvilinear transducer and a Affiniti 70 (Philips Healthcare, Bothell, WA) with a 1–5-MHz convex probe. This examination was conducted in accordance with the prenatal ultrasound examination guidelines of the Ultrasound Physicians Branch of the Chinese Physicians Association. In addition, the abnormalities of placenta, umbilical cord, amniotic fluid and other accessory structures were also observed carefully. A detailed fetal echocardiogram examination was performed according to the practice guidelines of fetal echocardiography issued by the International Society of Ultrasound in Obstetrics and Gynecology (Carvalho et al., 2013). Two dimension and color Doppler were used to evaluate the position, axis and size of the heart, the connection of atrium-ventricle-artery, and the return of systemic veins and pulmonary veins. The ultrasonographic findings of the fetus were as follows: (1) fetal growth restriction; (2) cleft lip and palate (Figure 2A); (3) bilateral renal hypoplasia; (4) lateral ventriculomegaly; (5) single umbilical artery; (6) absent stomach; (7) unroofed coronary sinus syndrome (Figure 2B); (8) coronary sinus dilatation with persistent left superior vena cava (Figure 2C); (9) membranous ventricular septal defect; (10) main pulmonary artery was dilated with inner diameter 7.6 mm, both branch pulmonary arteries arise from the right side of main pulmonary artery.

After genetic counseling, amniocentesis and CMA were performed at 26 weeks of gestation. The pregnant woman signed an informed consent and was fully informed of the risks of prenatal diagnosis, the advantages and limitations of testing methods.

The genomic DNA of amniotic fluid was digested, amplified, purified, fragmented, labeled, hybridized, washed and scanned strictly according to the standard protocol of the Affymetrix CytoScan platform (Affymetrix, Santa Clara, CA, USA), and the data were analyzed with ChAS 2.0 software. CNVs ≥ 50 Kb containing at least 20 contiguous markers were called for further analysis, and public reference databases such as Clingen, OMIM, DGV, DECIPHER, ISCA, USCS and PubMed were searched for comprehensive analysis. According to the standards and guidelines released by the American College of Medical Genetics (Kearney et al., 2011), CNVs can be classified as pathogenic, likely pathogenic, likely benign, benign or variants of uncertain significance. The CMA results of the fetus (Figure 3) revealed a 28.025-Mb deletion at 1q23.3q31.2 containing 115 OMIM genes (chr1:164,559,675–192,584,768), which was classified as pathogenic. The pregnant woman ultimately chose to terminate the pregnancy at 28 weeks of gestation and underwent induced labor. The autopsy after induced labor confirmed the prenatal ultrasound findings.

## Discussions

CMA of this fetus revealed a 28.025 Mb deletion on chromosome 1q23.3q31.2 (chr1:164,559,675–192,584,768), which encompassed 115 OMIM genes. The size of deletions on chromosome 1q and the resulting phenotypes varied among patients. A previous case (Table 1) of del(1)(q23.3q31.1) showed similar phenotypes, which included fetal growth restriction, cleft-lip and cleft-palate, absent stomach, bilateral renal hypoplasia, lateral ventriculomegaly, and ventricular septal defect (Lee et al., 2018). However, unroofed coronary sinus syndrome detected by prenatal ultrasound was not reported before. Unroofed coronary sinus (UCS) refers to the communication between the coronary sinus wall and the left atrium, which is often associated with the persistent left superior vena cava (PLSVC). In fact, UCS is a special type of atrial septal defect, which is easy to be missed due to the lack of specific clinical features (Tonni and Grisolia, 2020; Khadkikar et al., 2021). The diagnosis of the lesion is highly significant for the patient's prognosis because it can result in pulmonary hypertension, brain abscess or cerebral embolism (Bonardi et al., 2012). PLSVC is the most common systemic venous abnormality, accounting for 0.5% of the general population, and 3–8% of people with congenital heart disease (Galindo et al., 2007). The fetus with PLSVC is often associated with intra-cardiac and/or extra-cardiac anomalies. Ventricular septal defect, endocardial cushion defect and tetralogy of Fallot are common intra-cardiac malformations, while single umbilical artery is a common extra-cardiac malformation (Berg et al., 2006). Intra-cardiac and/or extra-cardiac anomalies in our patient were demonstrated by ventricular septal defect and single umbilical artery. In addition, PLSVC is more common in fetuses with chromosomal abnormalities, and PLSVC fetuses with other structural malformations have a higher correlation with chromosomal diseases than fetuses with isolated PLSVC (Du et al., 2014), so CMA should be recommended.

**Table 1:** Genotypes and phenotypes of the 20 previously identified cases of 1q23–32.1 deletions.

**Reported case**	**Cytoband**	**Deletion size (Mb)**	**GR**	**PR**	**L/P**	**GA**	**H/F**	**BD**	**CL**	**CA**	**MC**	**MG**	**RA**
Present case	1q23.3–q31.2	28.025	+	NA	+	-	-	-	-	+	-	-	+
Sun et al. (2019)	1q23.3	1.871	-	-	-	-	-	-	-	-	-	-	+
Lee et al. (2018)	1q23.3–q31.1; Xp22.31	28; 1.68	+	NA	+	-	+	+	+	+	+	-	+
Hu et al. (2013)	1q25.2–q31.3	20.5	-	+	+	-	-	-	+	-	-	-	-
Milani et al. (2012)	1q31.1–q32.1	15.6	-	+	+	-	-	-	-	-	-	-	-
Filges et al. (2012)	1q25.2–q25.3	1.5	+	-	-	-	-	-	-	+	-	-	-
Burkardt et al. (2011), P1	1q24.3–q25.2	12.48	+	+	-	+	+	+	+	-	+	-	-
Burkardt et al. (2011), P3	1q24.1–q31.1	26.7	+	+	+	+	-	+	-	+	+	-	-
Burkardt et al. (2011), P6	1q24.3–q25.3	9.81	+	+	+	-	+	-	+	-	+	+	-
Burkardt et al. (2011), P7	1q24.3–q25.3	12.9	+	+	-	+	+	+	-	-	+	-	-
Burkardt et al. (2011) P9	1q24.3–q31.3	22.3	+	+	+	+	+	-	+	-	+	+	-
Prontera et al. (2011)	1q24.3–q31.3	21	+	NA	-	-	-	-	-	-	+	-	NA
Nishimura et al. (2010)	1q24.3–q31.2	19.5	+	+	+	+	+	+	+	-	+	-	-
Thienpont et al. (2009)	1q25.1–q31.3	20.3	+	+	+	-	+	-	+	-	-	-	-
Descartes et al. (2008)	1q23.3–q25.2	14.38	+	+	+	+	+	+	+	+	+	+	-
Chaabouni et al. (2006)	1q24.2–q25.2	10.8	+	NA	-	-	-	+	-	-	-	-	-
Höglund et al. (2003)	1q25.3–q31.3	13.1	-	+	-	+	-	+	-	-	-	+	-
Pallotta et al. (2001)	1q23–q31.2	NA	+	+	+	-	+	+	+	-	+	-	-
Takano et al. (1997)	1q24–q25.3	NA	+	+	+	+	-	-	+	+	-	-	NA
Taysi et al. (1982) (P2)	1q23–q25	NA	+	+	+	+	+	-	+	+	+	-	-

*Phenotypes of each deletion are listed in the box. Adapted from Hu et al. (2013). GR, growth retardation; PR, psychomotor retardation; L/P, lip/palate anomalies; GA, genital abnormalities; H/F, small hands/feet; BD, brachydactyly; CL, fifth finger clinodactyly; CA, cardiac anomalies; MC, microcephaly; MG, micrognathia; RA, renal abnormalities; +, positive; –, negative; NA, no available; P, patient*.

The abnormal expression of *PBX1* have been reported to be associated with congenital heart disease (CHD) previously (Alankarage et al., 2020). Except for three cases reported by Hoglund, Milani and Hu (Höglund et al., 2003; Milani et al., 2012; Hu et al., 2013), severe pre- and/or postnatal growth retardation was found in almost all the patients with 1q23.3-q32 deletion. *PBX1, LHX4* and *CENPL* remain to be the primary candidate genes for growth retardation.

*PBX1* regulates numerous embryonic processes, including morphologic patterning, organogenesis and hematopoiesis (Ficara et al., 2008). *PBX1*, which is most strongly expressed in fetal kidney and brain, is an important regulator of interstitial function in renal morphogenesis and plays a key role in interstitial-epithelial signal transduction (Le Tanno et al., 2017). Patients with pathogenic *PBX1* mutations/microdeletions showed multiple developmental defects similar to those in *PBX1*^−/−^ mice, including external ear malformation, abnormal branchial arch derivatives, heart malformations, diaphragmatic hernia, renal hypoplasia and ambiguous genitalia (Slavotinek et al., 2017). Zhou et al. found that *PBX1* was a unique functional transcription factor in dNK cells, which promoted fetal development by up-regulating the expression of GPFs. The lack of *PBX1*^+^ dNK cells led to fetal growth restriction (Zhou et al., 2020). *LHX4* gene is located on 1q25 and involved in the regulation of motoneuron localization and pituitary development. Haploin sufficiency of *LHX4* could lead to growth hormone deficiency in patients with a deletion of this area. Another gene, *CENPL* gene at 1q25.1 is important for proper kinetochore function and mitotic progression. The deletion of this gene would result in growth deficiency (Lam and Morris, 2016).

Fetal growth restriction (FGR) means that fetal growth does not reach its due genetic potential due to the influence of maternal, fetal, placental and other pathological factors, which is characterized by the fact that the fetal weight or abdominal circumference estimated by ultrasound is lower than the 10th percentile of the corresponding gestational age (Galan and Grobman, 2019). Based on the growth status of different gestational weeks, fetal weight can be estimated and dynamic monitoring can be used to understand the fetal growth trend. Detailed ultrasound scans of fetal structure are recommended for FGR.

Brenner et al. proposed that FGR caused congenital renal hypoplasia (Brenner et al., 1988). Renal hypoplasia was defined as abnormally small kidneys with normal morphology and reduced nephron number. Current clinical practice utilizes ultrasonography to measure fetal kidney size and echogenicity as a means of evaluating the severity of renal hypoplasia and dysplasia (Phua and Ho, 2016). Interestingly, the kidneys appeared uniformly echogenic on ultrasound without cysts, and were difficult to differentiate from normal kidneys. The renal artery flow on Doppler couldn't always be recorded. Decreased blood flow to fetal kidneys could be responsible for decreased renal volumes (Slabiak-Blaz et al., 2015). However, renal hypoplasia is more likely the consequence of a malformative embryologic process. *PBX1* was thought to play a crucial role during embryogenesis by regulating the expression of developmental genes thus influencing proliferation or remodeling processes (Le Tanno et al., 2017). Defects in the induction and patterning of the developing kidney can cause congenital anomalies of the kidney and/or urinary tract (CAKUT), a wide range of renal-related birth defects including kidney agenesis, kidney hypoplasia or dysplasia, horseshoe kidney, cystic kidneys, or duplex kidney, as well as multiple collecting ducts or ureters abnormalities (San Agustin et al., 2016). Efforts to systematically elucidate genetic link between CAKUT and CHD have been reported, suggesting that CAKUT is highly associated with CHD (Gabriel et al., 2018). These findings may allow CHD fetuses benefit from early diagnosis and early therapeutic intervention of CAKUT. In addition, the mutations in ciliopathies also lead to kidney disease, which is called kidney ciliopathies. Unlike previous studies, we could not found any genes involved in ciliopathies or ciliogenesis in the deleted region (Table 2) (Devlin and Sayer, 2019).

**Table 2:** Summary of novel renal ciliopathy genes and their function. Adapted from Devlin and Sayer (2019).

**Gene**	**Cytogenetic location/Genomic coordinates**	**Encoding Protein**	**Protein function**
**Novel renal ciliopathy genes**			
ADAMTS9	3p14.1(chr3: 64,501,330-64,673,365)	A disintegrin-like and metalloproteinase with thrombospondin Type 1 Motifs 9	A member of the zinc-dependent ADAMTS metalloproteinases, important in the ER-Golgi transport system and extracellular matrix degradation. Localizes in close proximity to basal body. *In vitro* studies indicate a potential role in ciliogenesis/cilia maintenance and Hh signaling, but exact molecular mechanisms need to be determined.
ARL3	10q24.32(chr10: 104,433,484–104,474,190)	ADP ribosylation factor-like GTPase 3	Cilia GTP-binding protein, required for release of lipidated protein ciliary cargo. Crucial for ciliogenesis and formation of the axoneme.
CEP55	10q23.33(chr10: 95,256,369–95,288,849)	Centrosomal protein 55 kDa	CEP55 is a centriolar protein that localizes at the midbody and is required for abscission during cytokinesis. Potential regulator of the AKT/PI3K pathway. Any roles in cilia formation/maintenance/precneqqfunction is yet to be confirmed.
DZIP1L	3q22.3(chr3: 137,780,827–137,834,451)	DAZ interacting protein 1-like	Centriolar protein localizes to the mature centriole overlapping with the transition zone, subdistal and distal appendage markers. Potential roles in polycystin 1/2 cilial distribution, however exact molecular function needs to be confirmed.
OFIP(KIAA0753)	17p13.1(chr17: 6,481,645–6,544,247)	OFD1-AND-FOR20-interacting protein	Centriolar and centriolar satellite protein, component of the FOR20-OFD1-OFIP complex required for centriolar integrity, microtubule stabilization and centriole plasma membrane anchoring. *In vitro* studies indicate interactions with CEP63, which is important in centriolar duplication. Exact functions need to be confirmed.
TMEM260	14q22.3(chr14: 57,046,511–57,116,233)	Transmembrane protein 260	A predicted transmembrane protein, with two main isoforms. Its exact function is unknown.
TXNDC15	5q31.19(chr5: 134,209,460–134,237,323)	Thioredoxin domain-containing protein 15	Positive regulator of ciliogenesis, and predicted to interact with multiple cilia proteins, but no clear cilia localization. Positive regulator of Hh signaling. Molecular function needs to be confirmed.
**Novel renal ciliopathy candidate genes**			
RUVBL1	3q21.3(chr3: 127,783,628–127,872,757)	RUVB E.coli homolog-like 1 (Pontin)	An ATPase from the AAA + family of ATPases that functions in protein chaperone and co-chaperone complexes. It has roles in DNA Damage response pathways, mitotic spindle repair and chromatin remodeling. *In vitro* proteomic screens have shown Ruvbl1 is a cilia protein which interacts with the NPHP complexes in cilia, but Ruvbl1 does not have a ciliary localization. Its exact cilia function is unknown.
**Candidate renal ciliopathy modifier genes**			
ATMIN	16q23.2(chr16: 81,069,458–81,080,951)	ATM Interactor	Transcription factor, regulates ATM activity and stability in absence of DNA damage. ATMIN is required for Wnt-mediated signaling in kidney development. *In vitro* studies have demonstrated ATMIN influences Pkhd1 mRNA levels.
GLI1	12q13.3(chr12: 57,853,918–57,866,051)	Glioma-associated oncogene homolog 1	Zinc-finger transcription factor, regulated by Hh signaling. It is associated with stem cell proliferation, tumorigenesis, and numerous signaling pathways, including ERK and G-Beta Gamma.
NME3	16p13.3(chr16: 1,820,321–1,821,710)	Nucleoside diphosphate kinase 3	A nucleoside diphosphatre kinase that facilitates DNA-repair mechanisms and binds to NEK8, CEP164 and ANKS6.
**Novel non-ciliary genes associated with a renal ciliopathy phenotype**			
GANAB	11q12.3(chr11: 62,392,298–62,414,198)	Alpha-Glucosidase II	This gene encodes the alpha (catalytic) subunit of glucosidase II, an endoplasmic reticulum enzyme. *In vitro* studies indicate that GANAB effects polycystin 1 and 2 maturation and cilia localization. The exact pathogenesis and cilia function of GANAB are not known.
DNAJB11	3q27.3(chr3: 186,288,465–186,303,589)	DnaJ heat shock protein family member B11	Soluble glycoprotein of the endoplasmic reticulum. Interacts with BiP. Required for maturation process of polycystin-1.
MAPKBP1	15q15.1(chr15: 42,066,632–42,120,053)	Mitogen-activated protein kinase-binding protein 1	Scaffold protein for mitogen-activated protein kinases, which is key for regulating JNK and NOD2 signaling. Non-ciliary protein, but has a suggested MAPKBP role in DNA Damage Response pathways.
**Novel renal ciliopathy phenotypes for known ciliopathy genes**			
C2CD3	11q13.4(chr11: 73,723,759–73,882,064)	C2 calcium-dependent domain containing protein 3	Centrosomal cilia protein, that is vital for distal appendage formation, IFT component recruitment and ciliogenesis regulation.
CCDC114	19q13.33(chr19: 48,799,709–48,823,332)	Coiled-coil domain containing protein 114	Cilia protein that is predicted to be associated with the outer dynein docking arm complex along the axoneme. Is also localized to the basal body in some cell types.
CEP19	3q29(chr3: 196,433,148–196,439,165)	Centrosomal protein 19 kDa	Centrosomal protein that localizes to the primary cilia basal body; it is important for IFT-B recruitment into the primary cilium and subsequent ciliogenesis.
NPHP1	2q13(chr2: 110,880,913–110,962,639)	Nephrocystin-1	Cilia protein, interacts with nephrocystin-4 as well as functioning at tight junctions with a role in epithelial cell organization.
IFT140	16p13.3(chr16: 1,560,428–1,662,109)	Intraflagellar Transport 140	A component of the IFT-A complex, which is involved in cilia intraflagellar transport.
IFT27	22q12.3(chr22: 37,154,246–37,172,177)	Intraflagellar Transport 27	IFT27 is a g-protein that forms a component of IFT-B complex, which is required for cilial intraflagellar transport.
RPGRIP1L	16q12.2(chr16: 53,633,151–53,737,850)	RPGRIP1-Like	Cilial protein, localized at the basal body of the primary cilia, ciliary axoneme and cytoplasm. Interacts with nephrocystin 4, its exact function needs to be determined.

Our case is the first report of detailed prenatal ultrasound diagnosis of a fetus with del(1)(q23.3q31.2). When the fetus with FGR is complicated with abnormal ultrasound soft markers or structural abnormalities in the second trimester, interventional prenatal diagnosis and CMA are recommended. CMA is performed either by array comparative genomic hybridization or single nucleotide polymorphism array. Compared with classic cytogenetic and fluorescence *in situ* hybridization (FISH) techniques, the biggest advantage of using CMA for prenatal genetic diagnosis of chromosomal abnormalities is that CMA can detect much smaller imbalances (Levy and Wapner, 2018). Conventional G-banded karyotype analysis can only be able to detect the deletions or duplications over 5–10 Mb in size. The meta-analysis of the application of CMA to FGR compared with typical G-banding karyotype analysis showed that when FGR was associated with structural abnormalities, CMA could detect an additional 10% of the pathogenic CNV (Borrell et al., 2018).

## Conclusion

In this study, we reported a detailed description of the phenotypes in a fetus with del(1)(q23.3q31.2) and proposed some phenotype-related candidate genes. However, this deletion region didn't contain previously reported ciliary genes. Identification of additional affected fetuses with similar deletion of chromosome1q23.3–q31.2 is needed to provide further insights into the pathogenesis of 1q23.3–q31.2 deletion.

## Data Availability Statement

The genotyping data for this article are not publicly available to assure patient confidentiality and participant privacy. Requests to access the datasets should be directed to Ting Wang, biowt@njmu.edu.cn.

## Ethics Statement

The studies involving human participants were reviewed and approved by The Institutional Ethics Committee of The Affiliated Suzhou Hospital of Nanjing Medical University. Written informed consent to participate in this study was provided by the participants' legal guardian/next of kin.

## Author Contributions

JS and QZ made substantial contributions to conception and design, acquisition, analysis and interpretation of the data, and involved in drafting the manuscript. BL, ZG, TW, HT, and JX made substantial contributions to the acquisition, analysis, and interpretation of the data. WJ and XD involved in critically revising the manuscript for important intellectual content and gave the final approval of the version to be published. All authors contributed to the article and approved the submitted version.

## Funding

This study was supported by grants from the Gusu Health Talent Training Program (GSWS2019056).

## Conflict of Interest

The authors declare that the research was conducted in the absence of any commercial or financial relationships that could be construed as a potential conflict of interest.

## Publisher's Note

All claims expressed in this article are solely those of the authors and do not necessarily represent those of their affiliated organizations, or those of the publisher, the editors and the reviewers. Any product that may be evaluated in this article, or claim that may be made by its manufacturer, is not guaranteed or endorsed by the publisher.
